# Healthcare staff fatigue: The unrecognised risk for patient safety

**DOI:** 10.1016/j.fhj.2025.100250

**Published:** 2025-04-10

**Authors:** Laura Pickup, Nancy Redfern, Emma Plunkett, Suzy Broadbent, Mark S. Young

**Affiliations:** a Laura Pickup, Head of Human Factors, University Hospital Bristol and Weston, Bristol, UK; b Consultant Anaesthetist, Newcastle upon Tyne NHS Foundation Trust, Newcastle upon Tyne, UK; c Consultant Anaesthetist, University Hospitals Birmingham NHS Foundation Trust, Birmingham, UK; d Human Factors Specialist, Broadbent HF, Leeds, UK; e Professor of Human Factors in Transport, University of Southampton, Southampton, UK

**Keywords:** Fatigue, Patient safety, Risk management

## Abstract

Fatigue among NHS staff is rarely acknowledged as a risk to the quality of healthcare delivery and patient safety. Other safety-critical industries have systematic approaches to managing and accounting for the known risks of staff fatigue within their organisations. These provide individual staff with clear expectations, and require an organisation-wide approach to fatigue risk management and national oversight by a regulatory framework. Fatigue in health and social care is not managed in the same way. This paper describes why fatigue is a risk to patient safety and the core components of fatigue risk management systems, and includes reflections from the authors’ experiences in healthcare. This paper makes the case for healthcare organisations to acknowledge and seek to pragmatically manage the risk of fatigue among healthcare staff to support patient safety.

## Introduction

There is an extensive body of literature documenting both the causes and adverse effects of fatigue on both cognitive and physical performance.^1^ Fatigue is a human physiological state which may result from a reduction in hours slept, poor-quality sleep or sleep deprivation from extended periods of wakefulness. It can also be caused or exacerbated by high workload intensity or duration and/or working against circadian rhythms (the timing of the individual’s biological clock within the brain).

Healthcare staff are as susceptible to fatigue as those in other safety-critical industries. As in other such industries, the need for a 24-hour healthcare service requires staff to work at any time of the day or night. Human physiology is well understood by clinical staff in the context of treating patients. Recognition and effective management of their own personal physiology may be less appreciated; specifically, how circadian rhythms influence the reliability of performance and the impact of sleep deprivation (prolonged wakefulness, loss of a whole night’s sleep) and sleep restriction (chronic reduction of sleep time) on the safety of staff and patients. Regardless of the reason, an absence of sleep will have an impact on healthcare staff cognition, physiology and behaviours.

Fatigue among healthcare staff therefore comes at a cost. A reduction in the quality and reliability of clinical decisions and the delivery of treatments have subsequent consequences for patient safety.^1^^,^^2^ The cost is also incurred by staff themselves, as fatigue is associated with greater risks for driving to and from work, long-term health effects, as well as for NHS organisations in the retention of sufficient qualified staff to provide safe care.^3^

The evidence on the direct link between healthcare staff fatigue and degradation in patient safety is growing, but the scale of the risk remains unacknowledged.^4^^,^^5^ and the management of fatigue is not prioritised. Long working hours, poor or irregular shift patterns and high intensity of workload are the cultural norm for many UK healthcare staff. As healthcare has no formal mechanisms to acknowledge the systemic factors that influence staff fatigue, nor any measure of how many errors and near misses occur because of staff fatigue, healthcare is unaware of the scale of the risk that it is accepting and the impact of fatigue on healthcare work and patient and staff safety. The risk surrounding fatigue among healthcare staff is highlighted by individual professional bodies,^6^, ^7^, ^8^, ^9^, ^10^ but without systematic management of the risk or regulation of suitable controls, there are anomalies in the approaches adopted.

Healthcare has made attempts to consider and adjust to the Working Time Regulations 1998.^6^^,^^7^^,^^8^ However, these efforts tend to propose limits on hours of work without a risk-informed approach to balance these limits with operational demands and existing workforce challenges.^6^^,^^9^ The limitation on working hours alone is insufficient to reduce risk without considering operational factors such as staff workload and skill mix, as well as measures that may reduce the risk of fatigue such as regular breaks, rest facilities and evidence-based management and design of shifts, work patterns and staffing levels.^2^

There is a noticeable difference in maturity surrounding awareness and willingness to consider the risk of fatigue in healthcare compared to the formalised systems of fatigue risk management in other high hazard industries. The energy sector, rail and aviation industries all have evidence-based, funded and regulated fatigue risk management strategies and guidance.^11^, ^12^, ^13^

In 2018, Noone and Waclawski^14^ called for the need for healthcare to develop a comprehensive fatigue risk management system (FRMS), but in the UK this is yet to happen. Only one government can be found to have instigated this approach.^15^ This paper explains why fatigue should be considered as a risk for patient safety, introduces the principles and core components of FRMS based on scientific evidence, and shows how practitioners are applying and embedding such principles in other industries. We aim to demonstrate how a pragmatic approach to the implementation of FRMS could support UK healthcare to become aligned with other safety-critical industries.

## Why is fatigue a problem for patient safety?

Fatigue causes a degradation in cognitive performance, including slower response times, lapses in attention, reduced reliability in switching between tasks and worsening ability to reliably recall, manipulate and apply information to solve problems,^1^ It can also lead to increased risk-taking behaviour, including delays to decision-making and greater impulsiveness or adoption of strategies that prioritise short-term gains over longer-term success. The healthcare evidence on the effects of fatigue ranges across healthcare disciplines, types and severity of incidents, but includes prescribing,^16^ surgical^17^ and anaesthetic events.^18^ Furthermore, mortality rates have been shown to be related to staff fatigue levels.^19^ More broadly, fatigue may impact on teamworking and communication with patients as it reduces an individual’s ability to self-regulate, cope and demonstrate empathy towards others.

The potential consequences of fatigue also extend to staff safety, particularly when driving to and from work. Dawson.^20^ established that, after 17 h of being awake, our reaction times are equivalent to being at the limit of the alcohol level for driving in most western countries. After 24 h awake, our level of impairment is beyond the alcohol level limit for driving in the UK.^21^ Consequently, commuting to and from work and a 12-hour shift (that typically may extend beyond 12 h) could potentially exceed 17 h, and a clinician doing a 24-hour on call who is working at night is very likely to exceed this limit. Staff may be particularly susceptible at the end of a first night shift, where sleep may be difficult to obtain the day before. Concerningly, having less than 5 hours’ sleep is now recognised as doubling the risk of a driving incident.^22^

Fatigue may also impact the emotional state and perceptions of an individual, leading to an increase in negative emotions, depression, stress, anxiety and paranoia.^23^ Longstanding knowledge also exists around the effects of shift work and fatigue on the cardiovascular system, central nervous system and ocular system.^1^ If these are not managed, they have the potential to negatively influence both health, performance and sustainability of the healthcare workforce and retention of staff within healthcare organisations.

The financial implications of staff fatigue are a further reason for healthcare organisations to consider the importance of managing this risk. The costs associated with a fatigued workforce are not easy to capture, as few healthcare organisations identify when staff fatigue has been implicated in clinical errors. However, an international study suggested that 35% of people in the UK obtained <6 h sleep at a cost to the UK economy of £50 billion (GBP) a year. This was based on figures of absenteeism, presenteeism and reduced performance levels considered to reflect states of worker fatigue.^24^

In summary, there is strong evidence of the impact of fatigue on safety, performance, empathy and organisational finances. There is limited evidence, however, that healthcare formally recognises the risks posed by fatigue or takes steps to mitigate these risks. The next section describes the core components of one approach to manage the risk in the form of FRMS.

## The principles and components of a fatigue risk management system

Sprajcer *et al*^25^ described FRMS as ‘data driven management practices for the identification and management of risks related to fatigue’. They summarised that the emerging principles of FRMS across different industries indicated a trend for FRMS to be developed as a ‘flexible risk-based’ approach rather than prescriptive limitations on working times alone. The term ‘flexible’ may appear critical to industries that start from a baseline of low staffing numbers, historic practices of unregulated working hours or high demands on a service. The intention of a flexible, risk-based approach is to work within an organisation’s capability to identify, control and monitor the risks associated with fatigue. Fundamentally, the aim of FRMS is to maintain staff alertness and reduce the likelihood and impact of staff fatigue on safety-critical work.^21^ FRMS is intended to provide a coordinated organisational approach to managing the risk of fatigue and will typically have three core properties: predictive, proactive and reactive.^11^, ^12^, ^13^, ^25^ These core components are described in more detail in Table 1, in which the first column illustrates how they are typically applied in other industries.

**Table 1:** Summary of core components of a fatigue risk management system (FRMS) in the context of healthcare

Components of FRMS	Operational and organisational factors influencing FRMS for UK healthcare
**Predictive properties** The predictive properties of FRMS enable an organisation to anticipate fatigue-inducing conditions for specific job roles and to understand the implications of fatigue when modifying work practices. The organisation will consider and act upon factors known to impact sleep and fatigue in advance of daily operations^28^.	
**Working hours** Set parameters for shift duration and additional hours.	• Bank, agency, locum and permanent healthcare staff may have different hours of work and technical systems to record these. This can challenge the identification and analysis of total hours worked. • Financial imperatives for the employee may create a reluctance to share their total hours worked with managers. • Organisational financial incentives may influence extended working hours. • Variability in the design of systems used to monitor and manage work durations and individual staff health for different job roles.
**Sleep disorder screening** Identification of existing sleep disorders, eg sleep apnoea or insomnia.	• The absence of a national requirement to screen healthcare staff for sleep disorders requires organisations to recognise the value of identifying and supporting those with sleep disorders. • Occupational health or wellbeing checks with staff may not optimise the opportunity to identify sleep issues.
**Rostering** Evidence-informed rules on maximum number of consecutive duties, hours of work before a break and minimum duration of rest between duties including strategies to manage exceeding recommended work hours because of operational demands.	• For some staff groups, norms of shift scheduling in healthcare are not regulated according to evidence on fatigue and sufficiency of time to rest and recover. Where this is present (such as the Guardian of Safe Working in England), there can be inconsistencies in the use of these systems. • In some organisational cultures, operational demand and availability of staff are more likely to drive shift patterns, duration of work and breaks within and after shifts.
**Biomathematical modelling**^1^ Data-informed systems (based on principles of circadian rhythms) to support the prediction of fatigue-inducing work conditions; eg ratio of work hours, sleep times, commute times.	• Healthcare currently has limited data to develop this for all relevant job roles. • Inconsistency in use of technology to manage all staff work schedules.
**Proactive properties** Proactive properties of a FRMS seek to monitor, measure and manage fatigue levels during the day and as a consequence of current work design. This may include adjustments to schedules or fatigue countermeasures.^28^	
**Strategy** Organisational statement of commitment to manage risk of fatigue with a systematic approach.	Some healthcare organisations may have awareness of the impact of fatigue and aspects of FRMS, but there is currently little evidence of established FRMS in healthcare. Organisational readiness to identify and manage fatigue within the organisation’s risk register.
**Policy** Local departmental statements on management and mitigation of fatigue for specific contexts.	• Some healthcare policies indicate the need to consider fatigue, but currently healthcare does not have a standardised approach and policies on fatigue management are not required or reviewed by regulators. • Organisational policies that primarily seek to prevent fatigue and consider circadian physiology to mitigate risks of fatigue, for example napping on breaks or before driving.
**Roles and responsibilities** Descriptions of stakeholders and allocation of responsibilities, such that FRMS is implemented and reviewed.	• The roles and responsibilities for FRMS need to be established within healthcare; however, these will vary across organisations to reflect differences in organisational structures.
**Education** Promotion, training and awareness of the risk of fatigue.	• Limited awareness raising about the risk of fatigue and its impact on patient and staff safety, education about strategies for mitigation, promotion of fatigue-aware clinical practice. • Communication on organisational approaches vary in the priority placed upon the management of fatigue. Some regard it as essentially an individual responsibility, whereas those with more effective systems recognise the importance of team and organisational responsibility that is effectively communicated to staff. • Some national materials exist to support education on fatigue for different healthcare professionals but typically these emphasise individual responsibility and focus on good sleep hygiene.
**Rest facilities** Suitable break areas and facilities to support napping.	• Promotion and culture of organisation will vary in acceptability and prioritising availability and use of rest facilities to support napping to mitigate risks associated with fatigue. • Organisational infrastructure may impact availability, suitability and proximity to workplace of rest facilities.
**Organisational culture** Organisation procedures and attitude to embed management, self-assessment and reporting on fatigue.	• Culture within UK healthcare may impede adoption of recognised strategies to manage fatigue, ie controlled rests, napping during breaks. • Limitations in the organisation’s estate and prioritisation in use of space may impact provision of suitable facilities to support culture of prioritising rest. • Polices and leadership will influence a positive culture to promote reporting on fatigue. • Organisational approaches vary in the priority placed upon management of fatigue. Some regard it as essentially an individual responsibility, whereas those with more effective systems recognise the importance of team and organisational responsibility that is effectively communicated to staff.
**Reporting, measuring and monitoring** Individual and organisational tools.	• The design and inclusion of fatigue is not typically found within organisational reporting systems. • Limited resources and communication to support use of individual sleep diaries and recognised rating scales on fatigue. • Variability in the availability and organisational approach to use of physiological measures that indicate fatigue. • Lack of established data reporting which can capture and reflect fatigue-inducing conditions .
**Processes for management of fatigue** Established risk management process.	• Absence in prioritisation and coordination of processes across the organisation that support the identification, assessment, control and evaluation of known risks of fatigue.
**Reactive properties** Reactive properties of FRMS are intended to provide learning and identify the need to respond to the impact of fatigue, whilst enabling the organisation to continually improve.^28^	
**Incident investigation** Fatigue evidence considered within investigations.	• Limited use of systems and processes capable of identifying the impact of shift patterns, opportunities for rest and support to alleviate the impact of fatigue on performance.
**Audit** Continual evaluation of management of fatigue.	• Limited use of a continuous improvement approach to monitor reporting and management of fatigue within the organisation.
**Response** Contingency plans to respond to fatigue.	• The absence of a risk management approach to staff fatigue may prevent an organisational plan or process to manage staff on shift when fatigue is identified or declared.

1 Biomathematical models are scientific tools that help predict how work schedules might lead to fatigue. They are based on our natural sleep-wake cycle (circadian rhythm) and can estimate when staff are likely to be tired, when they might sleep, and how much rest they will get. These models can be useful for planning work shifts and reducing fatigue risks^12^.

### Effectiveness and implementation of FRMS

The adoption and implementation of FRMS varies in its maturity and approach across industries. Aviation has the longest history, starting in 1950, when the International Civil Aviation Organisation (ICAO) required airlines to limit flight times so as not to endanger the lives of the crew.^12^ Initially the approach was restricted to the prescriptive limitation of working hours, but later matured to a flexible risk-based approach.^11^ This reflects the wider evidence base on the effectiveness of managing fatigue in the form of FRMS across different industries. One such review considered five different industries, including healthcare and nursing staff.^25^ The findings suggested that FRMS had been shown to demonstrate improvement in staff alertness and psychomotor vigilance tasks, reduced accidents and absenteeism, and reported improvements in quality of sleep.

There is a consensus that, for the implementation of FRMS to be successful, it should be evidence-based but pragmatically reflect organisational and operational experience:^11^, ^12^, ^13^ one size does not fit all. Healthcare systems are complex, and this complexity will require healthcare to tailor FRMS to reflect the differences across a single organisation. The evidence also indicates that the development of FRMS should recognise the size of the organisation, limitations in its resources and be built on the fundamental principle of a shared responsibility at organisational and individual level.

Pragmatism in the implementation of FRMS can be seen in the rail industry; following the Clapham Junction disaster, the enquiry established fatigue in signalling technicians as a major contributor.^26^ The initial working time limits recommendation of no >72 h of work a week and no >13 duties in 14 days were not based on good risk management practices; rather, they were all that were pragmatic and operationally achievable at that time, such was the state of shiftwork practice before the accident. Since the accident, the rail industry and its regulator have moved towards a more comprehensive approach, aligned with the scientific evidence, which considers shift patterns, fatigue education, on-shift mitigations, reporting and investigation.^13^

FRMS is often considered as the gold standard for industries to achieve robust management of fatigue. However, evidence also suggests that this may be a challenge for many industries or organisations where there is an absence of a mature safety culture or established safety management system.^25^ Table 1 highlights several factors which suggest that healthcare may currently find this challenging.

In light of this and the current context of limited resources and capacity, a pragmatic risk-based approach would seem most appropriate for healthcare.^27^ This could be implemented by the organisation acting upon breaches in predefined working times; ie access to rest breaks, duration of time off between shifts, or a maximum number of shifts worked within a set timeframe.^25^

In Table 1, the second column presents organisational and operational factors influential to introducing the core elements of fatigue risk management into healthcare. These represent the authors’ reflections on where pragmatism is most likely to be required within the NHS.

## Conclusion

Healthcare organisations should acknowledge and seek to manage the risks of fatigue. The scientific evidence is clear and the link to patient safety is evident. Healthcare lags behind other industries in adopting a systematic approach to managing the risk of staff fatigue. One size does not fit all; healthcare can learn from the pragmatism adopted by other safety critical industries in implementing a system to manage the risk of fatigue, which recognises existing constraints. This paper presents the generic components of fatigue risk management systems (FRMS) and draws on the authors’ experience within healthcare and investigations.

The current risk of staff fatigue in healthcare will remain silent until a system is introduced to understand and manage the risks and recognise how this contributes to patient harm and the retention of a skilled workforce.

## CRediT authorship contribution statement

**Laura Pickup:** Writing – original draft, Writing – review & editing, Conceptualization. **Nancy Redfern:** Writing – review & editing. **Emma Plunkett:** Writing – review & editing. **Suzy Broadbent:** Writing – review & editing. **Mark S. Young:** Writing – review & editing.

## Declaration of competing interest

The authors declare that they have no known competing financial interests or personal relationships that could have appeared to influence the work reported in this paper.
